# Supplementary material for: Mutations in PCYT2 disrupt etherlipid biosynthesis and cause a complex hereditary spastic paraplegia
Source: Brain. 2019 Oct 22;142(11):3382–97. doi: 10.1093/brain/awz291 (PMC6821184; doi:10.1093/brain/awz291)
Supplement: awz291_Supplementary_Data [file awz291_supplementary_data.zip › awz291-Suppl_data/Supplementary_Data4.zip!/Supplementary information 3 - fibroblast lipidomics.zip!/Fibroblast lipidomics/Controle vs Patient2/Statistical plots and data/TG[O] species/Volcano plot_alternative.pdf]

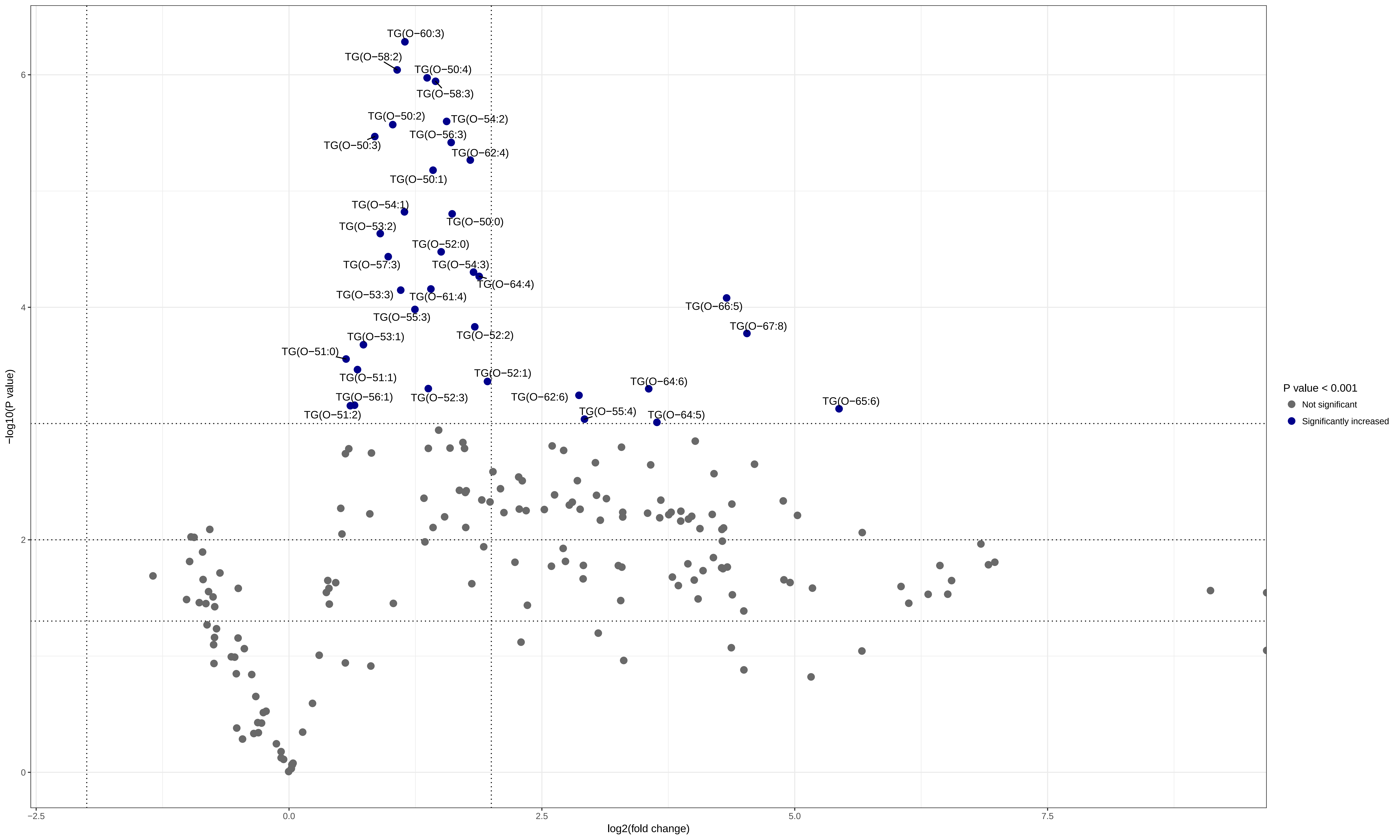

TG(O−60:3)
TG(O−58:2)
TG(O−50:4)
TG(O−58:3)
TG(O−50:2)
TG(O−54:2)
TG(O−56:3)
TG(O−50:3)
TG(O−62:4)
TG(O−50:1)
TG(O−54:1)
TG(O−50:0)
TG(O−53:2)
TG(O−52:0)
TG(O−57:3)
TG(O−54:3)
TG(O−64:4)
TG(O−53:3)
TG(O−61:4)
TG(O−55:3)
TG(O−66:5)
TG(O−67:8)
TG(O−52:2)
TG(O−53:1)
TG(O−51:0)
TG(O−51:1)
TG(O−52:1)
TG(O−64:6)
TG(O−56:1)
TG(O−52:3)
TG(O−62:6)
TG(O−65:6)
TG(O−55:4)
TG(O−51:2)
TG(O−64:5)
−log10(P value)
log2(fold change)
6
4
2
0
−2.5
0.0
2.5
5.0
7.5
P value < 0.001
Not significant
Significantly increased
